# Enhanced Corrosion Protection of Mild Steel Using a Sn–Graphene Oxide Composite Coating

**DOI:** 10.1021/acsomega.6c01083

**Published:** 2026-05-18

**Authors:** Filipe dos Santos Vita, Victor Magno Paiva, Natasha Midori Suguihiro, Eliane D’Elia

**Affiliations:** † Department of Inorganic Chemistry, 28125 Universidade Federal do Rio de Janeiro, Avenida Athos da Silveira Ramos, 149, Cidade Universitária, Rio de Janeiro 21941-909, Brazil; ‡ Department of Nanotecnology, Universidade Federal do Rio de Janeiro, Campus UFRJ−Duque de Caxias Professor Geraldo Cidade, Rodovia Washington Luiz 19593, Duque de Caxias 25240-005, Brazil

## Abstract

Sn–graphene oxide (Sn–GO) composite coatings were electrodeposited on mild steel to improve the corrosion resistance of tin-based protective layers. Graphene oxide was synthesized and characterized by X-ray diffraction (XRD) and Fourier transform infrared spectroscopy (FTIR), confirming its characteristic structural features and oxygen-containing functional groups. The electrodeposition parameters, including current density, graphene oxide concentration, and deposition time, were investigated using an experimental design methodology. The corrosion behavior of the coatings was evaluated by electrochemical impedance spectroscopy (EIS) and potentiodynamic polarization measurements in a 3.5% NaCl solution. The Sn–GO coating obtained under the best-performing conditions (0.125 g L^–1^ GO, current density of 10.0 mA cm^–2^, and deposition time of 30 min) exhibited improved morphology and enhanced anticorrosive performance compared with pure tin deposits. Electrochemical results showed a significant increase in charge transfer resistance, from 3834 Ω cm^2^ for metallic tin to 13149 Ω cm^2^ for the Sn–GO composite coating. These results demonstrate that the incorporation of graphene oxide, combined with appropriate electrodeposition parameters, leads to composite coatings with improved barrier properties and corrosion resistance, highlighting the potential of Sn–GO coatings as promising protective materials for industrial applications.

## Graphical abstract

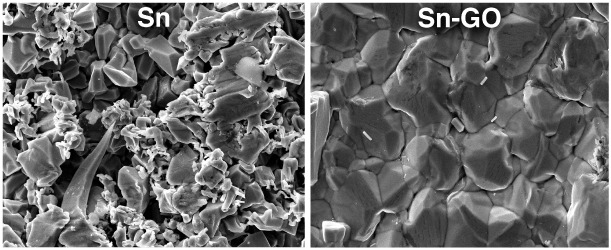

## 1 Introduction

Corrosion is a major challenge for modern society, causing significant economic losses and compromising the durability of metallic structures used in industrial, infrastructure, energy, and transportation sectors. As a spontaneous electrochemical process, corrosion leads to the progressive degradation of metals when exposed to aggressive environments, requiring the development of efficient protection strategies. According to studies conducted by the International Zinc Association, corrosion-related losses can represent a substantial fraction of national economies.^1^ Corrosion represents a major economic challenge worldwide, with estimated global costs corresponding to approximately 3–4% of the gross domestic product (GDP), highlighting the importance of developing effective corrosion prevention strategies.^2^

Various strategies have been proposed to mitigate corrosion, including the use of protective coatings, corrosion inhibitors, and the development of corrosion-resistant alloys. Among these approaches, anticorrosive coatings represent one of the most widely used methods because of their versatility and relatively low cost.^3^ Coatings can function as physical barriers that isolate the metallic substrate from the environment or act as sacrificial layers that preferentially corrode, thereby protecting the underlying material. Organic coatings such as paints are widely used; however, metallic coatings often provide superior durability and better mechanical and thermal resistance under harsh conditions.^4^

Among metallic protective coatings, tin (Sn) has attracted considerable attention due to its low toxicity, good corrosion resistance, and broad industrial applicability, including its use in electronics, food packaging, and soldering. Tin coatings can be readily produced by electrodeposition, enabling for precise control of coating thickness and morphology. However, despite their satisfactory performance, pure tin coatings may be limited in highly aggressive environments, which have motivated the development of composite coatings incorporating functional additives to enhance their protective properties.^5^

In recent years, graphene-based materials have emerged as promising nanomaterials for corrosion protection applications. Graphene oxide (GO), in particular, has attracted significant interest due to its high surface area, excellent barrier properties, and the presence of oxygen-containing functional groups that facilitate its dispersion in aqueous media and incorporation into composite coatings.^6–10^ Several studies have reported that the addition of graphene-based materials for protective coatings can significantly improve corrosion resistance by reducing coating porosity and hindering the diffusion of aggressive species, such as oxygen and chloride ions.^6–10^

Previous studies have demonstrated the potential of graphene oxide as an additive in electrodeposited metallic coatings. For instance, Gupta and Srivastava investigated the incorporation of graphene oxide into tin coatings electrodeposited on steel substrates and observed improved corrosion resistance at specific GO concentrations.^11^ However, their study was conducted under fixed electrodeposition conditions, without systematic optimization of the deposition parameters for pure tin coatings. This limitation restricts a comprehensive comparison between pure Sn coatings and Sn–GO composite systems and highlights the need for studies that explore the combined influence of electrodeposition parameters and GO incorporation on the properties of the resulting coatings.

Therefore, further investigations are necessary to better understand how electrodeposition parameters influence the formation, morphology, and anticorrosive performance of Sn–graphene oxide composite coatings. In this context, experimental design methodologies offer a useful approach for optimizing deposition conditions and identifying the key factors that affect coating performance.

In this work, the electrodeposition of pure tin and tin–graphene oxide composite coating onto mild steel substrates was systematically investigated. The effects of current density, deposition time, and graphene oxide concentration on the coating properties were evaluated using an experimental design. This study aims to develop Sn–graphene oxide composite coatings with enhanced corrosion resistance, thereby advancing more efficient anticorrosive systems for metallic substrates.

## 2 Materials and Methods

### 2.1 Synthesis of Graphene Oxide

The synthesis of graphite oxide in this study was based on the Hummers’ method. Initially, 1 g of graphite powder and 0.5 g of NaNO_3_ were added to a Kitasato flask placed in an ice bath. Subsequently, 25 mL of concentrated H_2_SO_4_ was gradually added with continuous magnetic stirring. The mixture was allowed to homogenize for 10 min, after which 10 g of KMnO_4_ was slowly introduced while stirring, followed by an additional 10 min to ensure complete homogenization.

After this step, the Kitasato flask was removed from the ice bath and heated to 35 °C on a hot plate for 3 h. Then, 50 mL of distilled water was carefully added, raising the temperature to approximately 98 °C; this temperature was maintained for 15 min under continuous heating. To terminate the oxidation reaction, a mixture of 166 mL of distilled water and 10 mL of 30-volume H_2_O_2_ was added to the reaction medium.

For purification of the graphite oxide, 60 mL of distilled water and 8 mL of concentrated HCl were added to the suspension. The mixture was then left undisturbed for 1 week to allow the graphite oxide to settle at the bottom of the flask. The supernatant was removed by decantation, and the solid was washed repeatedly with distilled water until the suspension reached approximately pH 5. The purified graphite oxide was collected on a watch glass, dried in an oven at 50 °C, and weighed.

To obtain graphene oxide, a dispersion was prepared by dissolving 1 g of graphite oxide in 140 mL of distilled water. The suspension was ultrasonicated for 20 h to promote exfoliation, and the resulting graphene oxide dispersion was stored for subsequent use.

### 2.2 Preparation of Mild Steel Plates

For subsequent coating, mild steel plates with the following nominal composition were used: 0.18 wt % C, 0.05 wt % P, 0.05 wt % S, 0.30 wt % Mn, and balance Fe (99.42 wt %).

Mild steel plates with nominal dimensions of approximately 18 mm in height, 25 mm in width, and 1 mm in thickness were used. Prior to electrodeposition, one face of the plate was isolated with an insulating coating, leaving a defined exposed area of approximately 0.8 cm^2^ for the electrochemical measurements. The exposed surface area was determined by measuring the electrode's height and width with a digital caliper.

Surface preparation consisted of manual grinding followed by abrading using an Arotec Aropol 2 V polishing machine. One face of each plate was ground only to 100-grit, whereas the opposite face—designated for metallic coating—was sequentially abraded using 100-, 320-, 600-, and 1200-grit abrasive papers.

After mechanical preparation, the samples were pretreated by immersion in a 20 wt % NaOH solution for 2 min, followed by immersion in 99% PA ethanol for an additional 2 min. Both steps were carried out under ultrasonication. The samples were then dried using interleaving paper, and the faces abraded only to 100 grit were coated with a colorless base enamel to electrically isolate them from the deposition process.

After drying at room temperature, the samples were immersed in a 5 wt % HCl solution for 30 s, rinsed, and dried again using interleaving paper. Finally, the height and width of the exposed electrode surface were measured by using a digital caliper, and the prepared samples were stored in a desiccator with interleaving paper until use.

### 2.3 Electrodeposition of Sn

After preparing the plates, 100.00 mL of the tin electrodeposition bath was prepared according to the composition proposed by Gupta and Srivastava (2018), as shown in Table 1.

**Table 1:** Composition of the Electroplating Bath of Metallic Tin

Composition	Concentration (g L^–1^)
Tin(II) sulfate	20.0
Sodium acetate	20.0
Sodium gluconate	140.0
Sodium lauryl sulfate	0.5

To prepare the tin electrodeposition bath, 2 g of tin­(II) sulfate was weighed and dissolved in a small volume of distilled water using an ultrasonic bath. Subsequently, 2 g of sodium acetate was added, followed by additional distilled water; after each addition, the mixture was briefly returned to the ultrasonic bath to ensure complete dissolution. Next, 14 g of sodium gluconate and 0.05 g of sodium lauryl sulfate were sequentially incorporated by using the same procedure. After all components had been added, the solution pH was measured (approximately 4), and the final volume was adjusted to 100.00 mL with distilled water in a volumetric flask.

Electrodeposition was carried out in a two-electrode system using an Autolab PGSTAT204 galvanostat/potentiostat (Metrohm). A pretreated mild steel substrate was used as the working electrode, while a platinum wire served as the counter electrode. Both electrodes were immersed in a beaker containing 50.00 mL of the prepared electrolyte, which was maintained under constant stirring using a magnetic bar. Film growth was achieved by chronopotentiometry, applying different current densities and deposition times according to the experimental design established using the Plackett–Burman model.

A Plackett–Burman (PB) experimental design was employed as a screening tool to evaluate the influence of electrodeposition parameters on the electrochemical performance of the coatings. For the tin coating, two independent variables were investigated: current density (A) and deposition time (B). Each factor was evaluated at two levels. In addition, three central points were included to estimate experimental variability and assess potential curvature effects.

In this study, the impedance modulus at a low frequency (|Z| at 0.01 Hz), obtained from electrochemical impedance spectroscopy (EIS) measurements after 24 h of immersion in a 3.5% NaCl solution, was used as the response variable. This parameter is widely recognized as a reliable indicator of the barrier performance and corrosion resistance of protective coatings since higher impedance values at low frequencies are generally associated with increased resistance to charge transfer and electrolyte penetration.^12–14^

Statistical analysis was performed using Action Stat software, and no factors were found to be significant within the investigated range. The best-performing deposition conditions were identified from the experimental run that yielded the highest impedance modulus at low frequency, corresponding to the highest corrosion protection performance among the tested coatings.

### 2.4 Electrodeposition of Sn–GO Composite Coatings

Electrodeposition baths for the tin–graphene oxide (Sn–GO) composite coatings were prepared using different graphene oxide concentrations and formulations either containing or excluding additives, as summarized in Tables 2 and 3.

**Table 2:** Composition of the Electrodeposition Bath of the Tin–Graphene Oxide Composite with Additives

Composition	Concentration (g L^–1^)
Tin(II) sulfate	20.0
Sodium acetate	20.0
Sodium gluconate	140.0
Sodium lauryl sulfate	0.5
Graphene oxide	0.125, 0.375, and 0.625

**Table 3:** Composition of Tin–Graphene Oxide Composite Electrodeposition Bath without Additives

Composition	Concentration (g L^–1^)
Tin(II) sulfate	20.0
Graphene oxide	0.125, 0.375, and 0.625

The preparation of the electrodeposition baths followed the same procedure as that used for the tin bath described previously. For baths containing additives, aliquots of the graphene oxide (GO) dispersion were added immediately after sodium lauryl sulfate was incorporated, and the final volume was adjusted to 100.00 mL with distilled water.

For baths without additives, the components of sodium acetate, sodium gluconate, and sodium lauryl sulfate were omitted. In this case, the GO dispersion was added directly after the dissolution of tin­(II) sulfate, and the final volume was similarly adjusted to 100.00 mL.

The electrolytic cell configuration was identical to that employed for pure tin electrodeposition. However, in this case, different GO concentrations were investigated, together with variations in current density and deposition time according to the experimental design based on the Plackett–Burman statistical screening model. Each factor was investigated at two levels. In addition, three central points were included to estimate experimental variability and assess potential curvature effects.

Film growth was achieved by chronopotentiometry using different GO concentrations, current densities, and deposition times, as defined by the experimental design. Statistical analysis was performed using Action Stat software, and no factors were found to be significant within the investigated range. The best-performing deposition conditions were identified based on the experimental run that yielded the highest impedance modulus at a low frequency, corresponding to the highest corrosion protection performance among the tested coatings.

After electrodeposition, the coatings were dried and conditioned following the same procedure adopted for tin coatings. To verify the reproducibility of the results, both Sn–GO composite coatings and pure Sn coatings were electrodeposited in duplicate under the optimal conditions identified through the experimental design.

### 2.5 Electrochemical Measurements

Glass electrochemical cells were assembled containing a 3.5 wt % NaCl solution, in which the coated samples were immersed and maintained in continuous contact with the saline medium for 24 h before electrochemical testing.

After approximately 23 h of immersion, a three-electrode system connected to a Metrohm Autolab potentiostat equipped with a PGSTAT128N frequency response analyzer was coupled to the electrochemical cell. A saturated calomel electrode (SCE) was used as the reference electrode, a platinum electrode as the counter electrode, and the coated sample—exposed on one side with an area of approximately 0.8 cm^2^—served as the working electrode. The assembled cell was placed inside an aluminum enclosure serving as a Faraday cage, and the open circuit potential (OCP) was monitored using NOVA 2.1.5 software until stabilization. Exactly 24 h after immersion, electrochemical impedance spectroscopy and potentiodynamic polarization measurements were initiated.

Electrochemical impedance spectra were recorded over a frequency range from 10 kHz to 10 mHz using a sinusoidal perturbation of 10 mV (rms) applied around the OCP. Potentiodynamic polarization curves were obtained by scanning the potential from −0.2 V to +0.2 V relative to the OCP at a scan rate of 0.166 mV s^–1^.

### 2.6 Material Characterization

Fourier transform infrared (FT-IR) spectra of graphite and graphene oxide were recorded using a Shimadzu IRAffinity-1 spectrometer equipped with an attenuated total reflectance (ATR) accessory and a ZnSe prism. Spectra were collected over the range of 4000–640 cm^–1^.

X-ray diffraction (XRD) patterns of graphite, graphite oxide, and graphene oxide were obtained using a Rigaku Ultima IV diffractometer with CuKα radiation (λ = 1.5406 Å, 40 kV, 100 mA) in the 2θ range of 5°–80°. Measurements were performed in step-scan mode with a step size of 0.02°.

Additionally, Sn and Sn–GO coatings deposited on mild steel substrates were examined by scanning electron microscopy (SEM) using a JSM-IT700HR instrument coupled to a JEOL energy-dispersive X-ray spectroscopy (EDS) system, operated at an accelerating voltage of 10 kV.

## 3 Results and Discussion

### 3.1 Synthesis of Graphite Oxide

The yields obtained for the graphite oxide syntheses are listed in Table 4. The experiments showed variable yields, with several values lower than those typically reported in the literature for the Hummers’ method. Reported yields often exceed 100%, indicating that oxygen atoms are incorporated into the graphite structure during oxidation.^15,16^

**Table 4:** Graphite Oxide Syntheses and Their Respective Yields

Synthesis	Graphite mass (g)	Graphite Oxide Mass (g)	Yield (%)
1	1.035	1.216	117.5
2	1.018	0.823	80.8
3	1.033	0.467	45.2
4	1.043	1.380	132.3
5	1.045	0.659	63.1

The lower yields observed in Table 4 are likely related to difficulties in controlling the temperature during the oxidation stage. Since the reaction is highly exothermic, the generated heat can promote overoxidation, leading to excessive CO_2_ formation and undesirable carbon loss.

The yield was calculated according to the equation below:

$$R\left(\%\right)=\frac{m_{ogr}}{m_{gr}}\times 100$$

where *m*_gr_ is the initial mass of graphite, *m*_ogr_ is the mass of graphite oxide obtained, and *R*(%) represents the yield.^17^

### 3.2 Characterizations of Graphite Oxide and Graphene Oxide

The graphite and graphene oxides were characterized by Fourier transform infrared (FTIR) spectroscopy using an attenuated total reflectance (ATR) accessory. The resulting spectra, as shown in Figure 1, display the characteristic features of each material.

**Figure 1:**
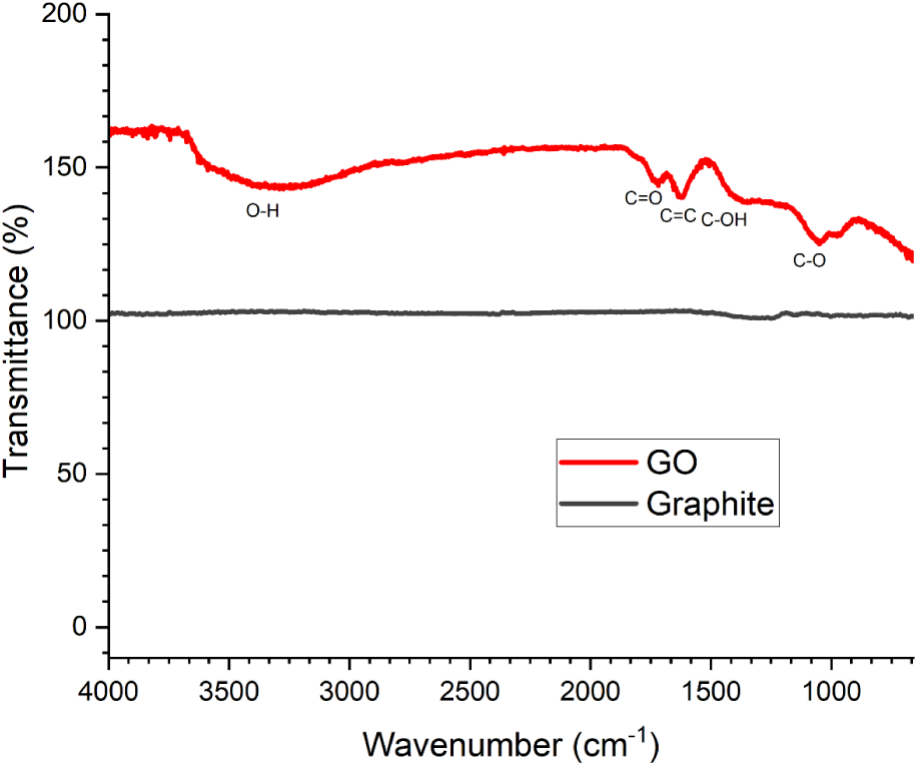
Fourier transform infrared (ATR) spectra of graphite and graphene oxide.

In the FTIR spectrum of graphene oxide (GO) shown in Figure 1, the broad band centered at 3293 cm^–1^ is attributed to the O–H stretching vibration, typically observed between 3200 and 3650 cm^–1^. The peak at 1701 cm^–1^ corresponds to the C=O stretching vibration from oxygen-containing functional groups, generally appearing in the range of 1700–1750 cm^–1^. Additionally, the bands at 1356 cm^–1^ and 1058 cm^–1^ are assigned to the C–O–H bending and the C–O stretching vibrations, respectively, which typically occur within 1220–1440 cm^–1^ and 1000–1300 cm^–1^, respectively.^16,18^

According to the literature, the peak at 1618 cm^–1^ in the GO spectrum is attributed to the C=C stretching vibration, which typically appears between 1600 and 1680 cm^–1^.^16,18,19^ However, this band may also correspond to the O–H bending vibration of adsorbed water molecules, which generally occurs around 1600 cm^–1^.^20^ In the graphite spectrum, no significant absorption peaks were observed, consistent with the high purity and chemical inertness of bulk graphite.^21^ A clear distinction between the FTIR (ATR) spectra of graphite and graphene oxide is evident, highlighted by the appearance of bands associated with oxygen-containing functional groups characteristic of GO, confirming the successful oxidation of graphite.^19^

Graphite and graphene oxides were further characterized by X-ray diffraction (XRD). The resulting diffractograms, shown in Figures 2 and 3, display the characteristic patterns of each material.

**Figure 2:**
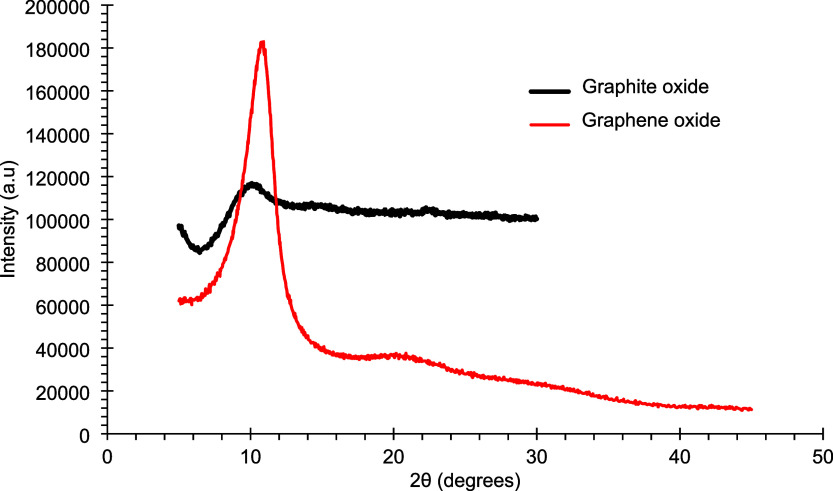
X-ray diffractograms of the synthesized graphite oxide and graphene oxide.

**Figure 3:**
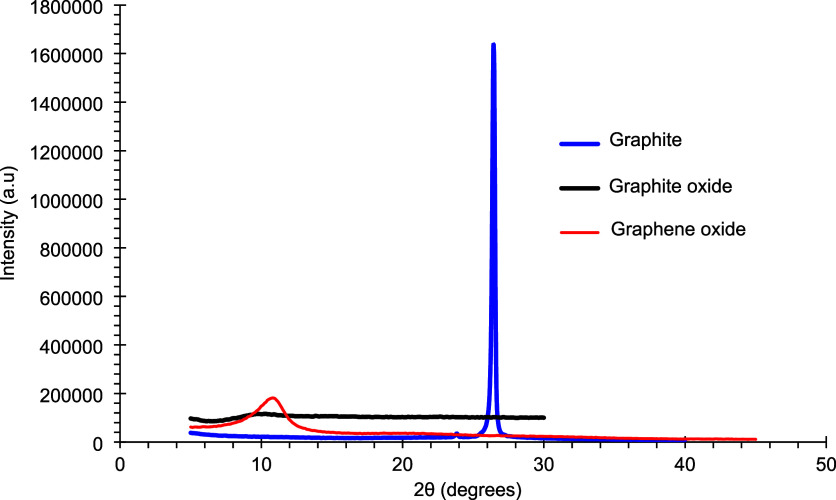
X-ray diffractograms of synthesized graphite, graphite oxide, and graphene oxide.

In Figure 2, a diffraction peak is observed at approximately 11°, characteristic of graphene oxide prepared via chemical oxidation. This peak reflects an increased in interlayer spacing relative to graphite, resulting from the intercalation of oxygen-containing functional groups between the graphene sheets. In Figure 3, the graphite peak appears at 26.5° (002), corresponding to the characteristic graphitic structure.^22–25^ Comparison of Figures 2 and 3 reveals the disappearance of the graphite peak, the appearance of a weak graphite oxide signal at a different position, and the subsequent emergence of the graphene oxide peak. This progression confirms the successful oxidation of graphite, in agreement with the FTIR results.

### 3.3 Evaluation of Tin Coatings

The coatings were electrodeposited using the chronopotentiometric technique at different current densities and deposition times, according to the Plackett-Burman statistical screening design, as summarized in Table 5.

**Table 5:** Experimental Planning of Metallic Tin Electrodeposition^a^

Plate	Time (min)	j (mA cm^–2^)
1	30	10.0
2	10	2.0
3	30	2.0
4	10	10.0
5	20	6.0
6	20	6.0
7	20	6.0

a Plates 5, 6, and 7 correspond to replicate experiments conducted at the central point of the experimental design.

The electrodeposition of tin occurs according to the following electrochemical half-reaction:

$${\mathrm{Sn}}^{2+}+{2e}^{-}\to \mathrm{Sn}$$

In general, the coatings were visually thin. They exhibited distinct appearances, as expected due to the different current densities and deposition times applied according to the experimental design shown in Table 5. In terms of color, deposit 5 exhibited a hue closest to the silver-white appearance reported in the literature, whereas samples 1 and 4 showed darker grayish tones. Deposits 6 and 7, although obtained under the same electroplating conditions as those for deposit 5, appeared darker and more matte. Moreover, the coating on plate 5 demonstrated the highest uniformity, while coatings 2 and 3 displayed larger defect areas. These variations can primarily be attributed to differences in current density and, possibly, to bath instability or the formation of undesirable species during the electrodeposition process.

The coated plates were subsequently subjected to electrochemical testing, including potentiodynamic polarization and electrochemical impedance spectroscopy, to evaluate their corrosion resistance. Before testing, the deposits were immersed in a 3.5% (m/v) NaCl aqueous solution for 24 h in an electrochemical cell, simulating a corrosive environment and allowing the corrosion potential to stabilize. The probable electrochemical reactions are indicated below:

$$\mathrm{Sn}\to {\mathrm{Sn}}^{2+}+{2e}^{-}$$

$$O_{2}+{2H}_{2}{O+4e}^{-}\to {\mathrm{4OH}}^{-}$$

Representative impedance diagrams and polarization curves were obtained for the coated samples, as shown in Figures 4–7 where −Z″ represents the imaginary component of impedance and Z′ represents the real component. In addition, the stabilized open-circuit potentials (OCP) were recorded and are presented in Table 6.

**Table 6:** Stabilized Open-Circuit Potentials (OCP) of Tin-Coated Plates after 24 h of Immersion in a 3.5% (m/v) NaCl Solution

Plate	Open Circuit Potential (V)
1	–0.694
2	–0.739
3	–0.717
4	–0.719
5	–0.607
6	–0.642
7	–0.684

**Figure 4:**
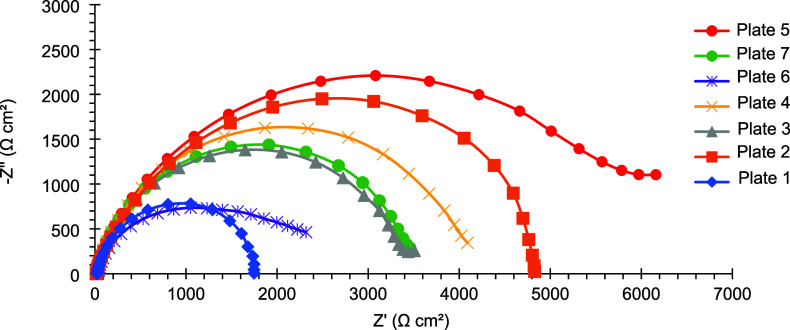
Nyquist diagrams of tin-coated plates 1–7 obtained after 24 h of immersion in a 3.5% (m/v) NaCl aqueous solution.

In the Nyquist diagrams shown in Figure 4, capacitive loops with different diameters are observed for each coating. These loops are associated with the charge transfer resistance (*R*_ct_) and the apparent double-layer capacitance (*C*_dl_), as described by the equivalent circuit model. A larger loop corresponds to a higher charge transfer resistance at the electrode–solution interface and, consequently, a lower corrosion rate.

Notably, plate 5 exhibited the largest capacitive loop among the samples, indicating superior corrosion resistance within the Sn-coating series. This result is consistent with the improved uniformity and brightness observed for this deposit, confirming its enhanced barrier properties against reactive species in a corrosive medium. In addition, as shown in Figure 5, coating 5 presented the highest impedance modulus in the low-frequency region (f = 0.01 Hz), further evidencing its effective barrier performance.^26^

**Figure 5:**
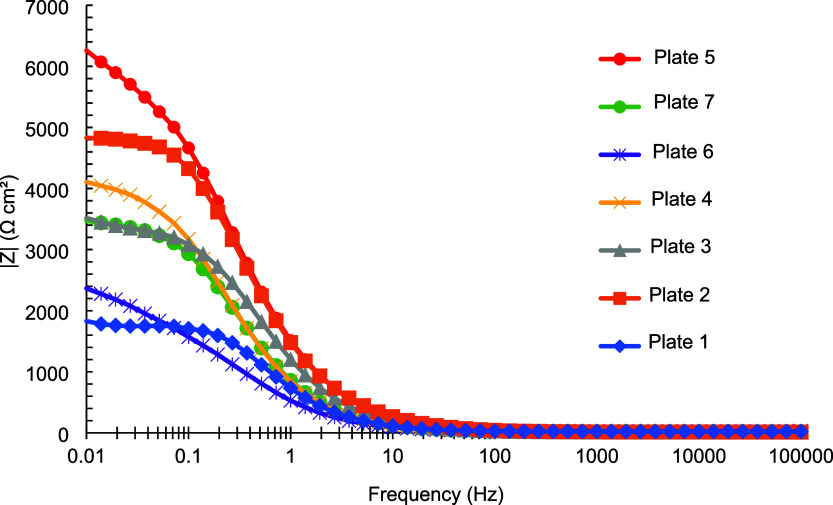
Bode plots of the impedance modulus for tin-coated plates 1–7 obtained after 24 h of immersion in a 3.5% (m/v) NaCl aqueous solution.

Moreover, Figure 6 shows that the same coating exhibited a maximum phase angle of approximately 70°, approaching the behavior of an ideal coating that acts as a nearly perfect capacitor at the electrode–electrolyte interface.^27,28^

**Figure 6:**
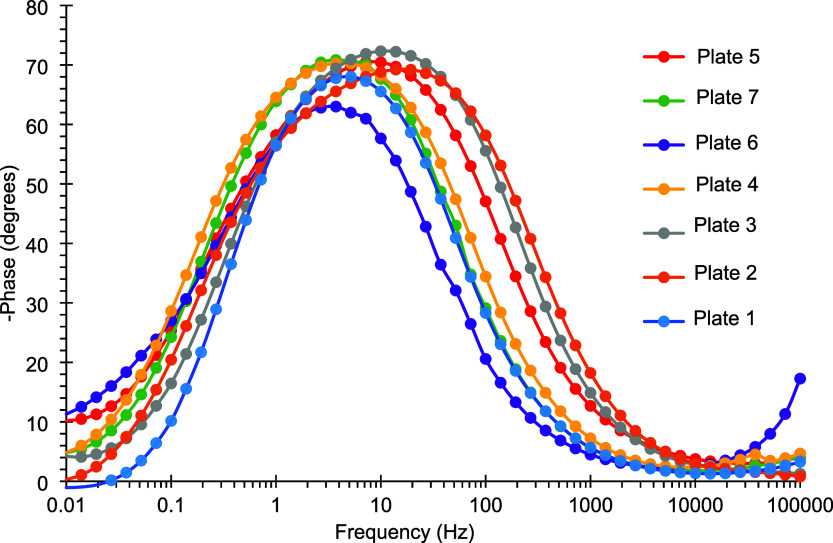
Bode plots of the phase angle for tin-coated plates 1–7 obtained after 24 h of immersion in a 3.5% (m/v) NaCl aqueous solution.

The other coatings exhibited lower to intermediate values of the impedance modulus and phase angle, consistent with their visual appearance. The specific values of these parameters for each coating, as determined according to the experimental design, are presented in Table 7.

**Table 7:** Impedance Modulus at 0.01 Hz and Maximum Phase Angle of Tin-Coated Plates after 24 h of Immersion in a 3.5% (m/v) NaCl Solution^a^

Plate	|Z|_0.01 Hz_ (Ω cm^2^)	–Φ_max_ (°)
1	1830	68
2	4828	69
3	3517	72
4	4105	70
5	6258	70
6	2364	63
7	3470	71

a The potentiodynamic polarization curves of the reduced Sn coatings on mild steel, following exposure to a 3.5% (m/v) NaCl corrosive medium, are presented in Figure 7.

There are several limitations to determining corrosion current density using the Tafel extrapolation method, including the requirement that the process involves a single cathodic reaction under activation control and that the linear regions in the log j versus E plot extend over at least one decade, among others. In the present study, the cathodic reaction is likely oxygen reduction, as suggested by the cathodic curve, which shows a broad region where the current density remains nearly constant—a hallmark of diffusion-controlled processes. Therefore, the Tafel extrapolation method was not applied, and the polarization curves were analyzed qualitatively instead.

Figure 7 shows that the polarization curve of plate 5 shifts tomore anodic potentials, accompanied by a marked decrease in anodic current density compared to the other coatings. A comparison between the anodic current densities and the corrosion potentials of the plates is listed in Table 8.

**Figure 7:**
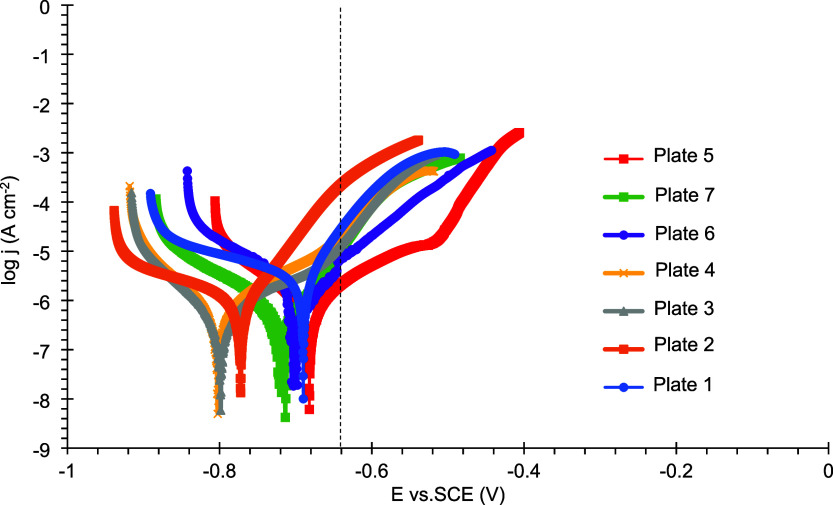
Polarization curves of tin-coated plates 1–7 after 24 h of immersion in a 3.5% (m/v) NaCl solution.

**Table 8:** Corrosion Potential of Tin-Coated Plates and Anodic Current Densities Obtained from Potentiodynamic Polarization after 24 h of Immersion in a 3.5% (m/v) NaCl Solution^a^

Plate	Corrosion Potential (V)	Anodic Current Density (mA cm^–2^)
1	–0.691	2.97 × 10^–2^
2	–0.773	2.29 × 10^–1^
3	–0.798	1.41 × 10^–2^
4	–0.803	2.10 × 10^–2^
5	–0.682	2.15 × 10^–3^
6	–0.701	7.19 × 10^–3^
7	–0.716	1.17 × 10^–2^

a Anodic current density values were obtained at −0.64 V.

According to Table 8, at −0.64 V, plate 5 exhibits the lowest anodic current density and, additionally, the highest corrosion potential (−0.682 V), indicating improved corrosion resistance. This behavior is consistent with the electrochemical results presented in Tables 6 and 7, where plate 5 exhibits the highest open circuit potential and impedance modulus at low frequency (0.01 Hz).

Based on the electrochemical results discussed previously, the impedance modulus data were subsequently analyzed using Action Stat software to evaluate the statistical significance of the parameters influencing the system. This analysis was conducted within the framework of a Plackett–Burman design, employed as an initial screening methodology to identify the most relevant parameters affecting the system with a reduced number of experiments.

The effects were calculated as the difference between the average responses at the high (+1) and low (−1) levels of each factor, as can be seen in Table 9.

**Table 9:** Plackett–Burman Design Matrix and Experimental Responses for Sn

Run	A	B	|Z|_0.01 Hz_ (Ω cm^2^)
1	10.0 (+1)	30 (+1)	1830
2	2.0 (−1)	10 (−1)	4828
3	2.0 (−1)	30 (+1)	3517
4	10.0 (+1)	10 (−1)	4105
5	6.0 (0)	20 (0)	6258
6	6.0 (0)	20 (0)	2364
7	6.0 (0)	20 (0)	3470
8	6.0 (0)	20 (0)	2917

Since the experimental design did not include replicates in the factorial portion, the statistical significance of the effects was assessed using Lenth’s method. The pseudo-standard error (PSE), margin of error (ME), and simultaneous margin of error (SME) were calculated at the 95% confidence level, as shown in Figure 8. The half-normal plot of the effects shows that all estimated effects align along the reference line and fall within the margin of error (ME), indicating that none of the factors are statistically significant. Specifically, the estimated effects for factors A (1205.13) and B (1793.31) were both lower than the ME, confirming that neither variable has a statistically significant influence on the impedance modulus within the investigated experimental domain. The responses at the central point (runs 5–8) are of the same order of magnitude as those of the factorial runs, indicating the absence of significant curvature in the response surface.

**Figure 8:**
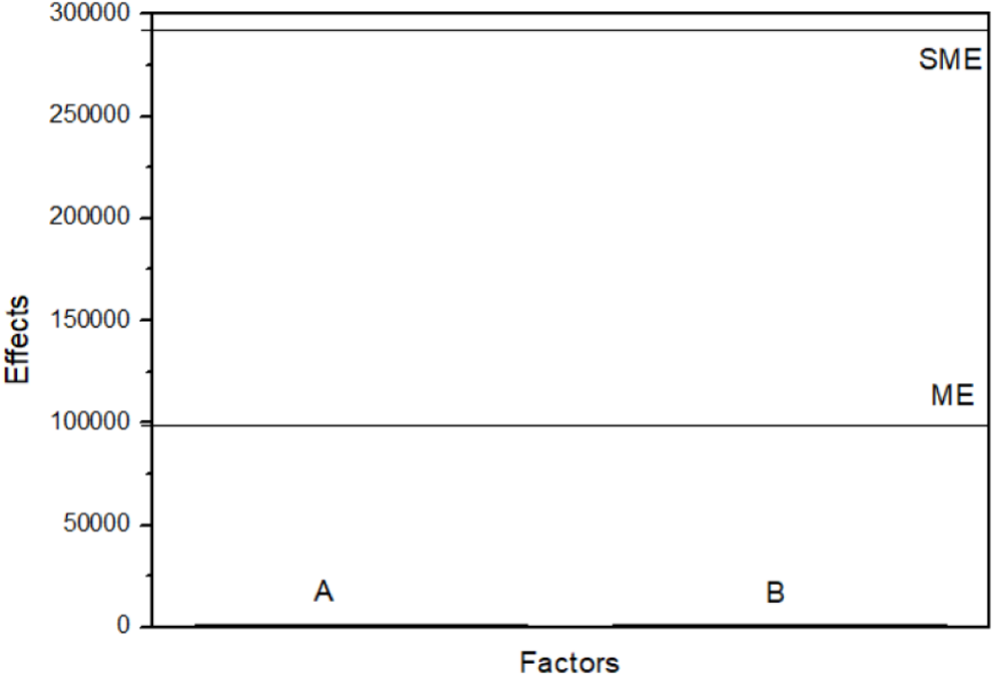
Half-normal plot of the effects for Sn.

As no statistically significant factors were identified, it was not possible to develop a predictive model for optimization based on the experimental design. Therefore, the optimal conditions were selected based on the experimental response values. The condition that yielded the highest impedance modulus was considered the most favorable within the studied range, as higher |Z| values are associated with improved corrosion resistance.

The experimental results indicated that the best response was obtained for plate 5 under conditions of j = 6.0 mA cm^–2^ and t = 20 min, consistent with the values reported by Gupta and Srivastava (2018) for tin electrodeposition. At a higher current density of j = 10.0 mA cm^–2^, the coating exhibited burning, as typically observed at elevated current densities, whereas at j = 2.0 mA cm^–2^, the deposits were fragile and of low apparent thickness, indicating an insufficient current density.

The morphology and microstructure of the best-performing coating were further investigated by using scanning electron microscopy (SEM). Figure 9 presents a representative micrograph of this coating.

**Figure 9:**
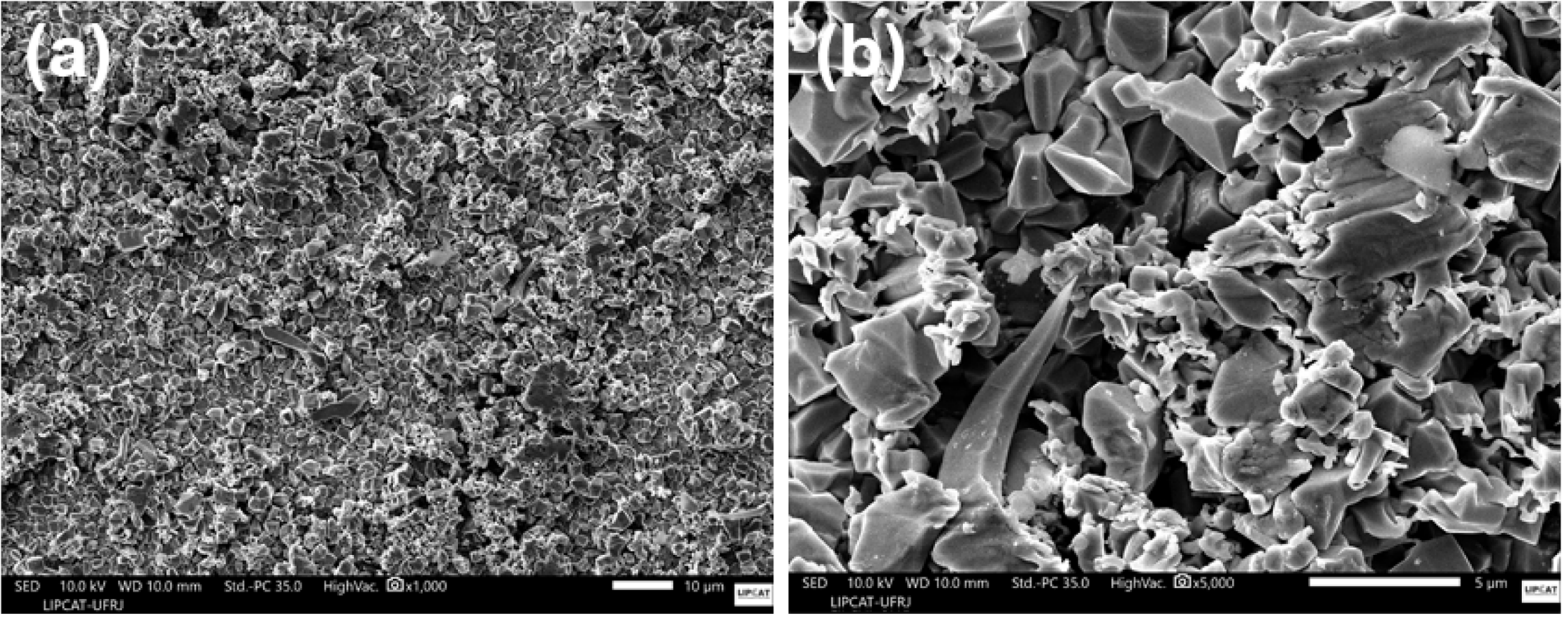
(a) Low-magnification and (b) high-magnification SEM micrographs of the Sn coating on plate 5.

The SEM image in Figure 9 shows that the coating on plate 5 exhibits a granular morphology with a polyhedral structure characterized by faceted, compact crystals arranged in dense agglomerates. The surface is rough, compact, and relatively homogeneous, suggesting a controlled crystallographic growth during electrodeposition. No significant porosities or large-scale cracks were observed. The coating composition in the same region was further analyzed by SEM-EDS, as shown in Figure 10.

**Figure 10:**
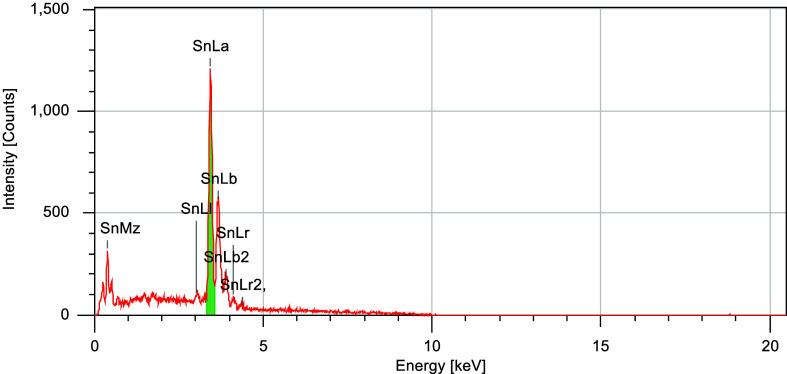
SEM-EDS analysis of the Sn coating on plate 5.

The composition was determined to be 100% Sn, in agreement with the SEM observations, confirming the successful electrodeposition of Sn onto the mild steel substrate.

### 3.4 Evaluation of Sn–GO Coatings

Composite coatings were electrodeposited using the chronopotentiometric technique under different current densities and deposition times, according to the experimental design established through the Plackett–Burman statistical screening model (Table 10).

**Table 10:** Experimental Design of Tin–Graphene Oxide Composite

Plate	j (mA cm^–2^)	GO concentration (g L^–1^)	Time (min)
1	10.0	0.125	10
2	2.0	0.625	30
3	6.0	0.375	20
4	2.0	0.625	10
5	10.0	0.625	30
6	6.0	0.375	20
7	10.0	0.125	30
8	2.0	0.125	30
9	10.0	0.625	10
10	6.0	0.375	20
11	2.0	0.125	10

In summary, the evaluation of additive effects indicated that coatings electrodeposited from baths containing additives exhibited improved morphological characteristics, including more uniform and compact deposits. SEM observations indicated that the presence of additives resulted in a more homogeneous coating with fewer visible defects, potentially improving interfacial contact with the substrate. This behavior can be attributed to the role of sodium gluconate as a complexing agent for tin, which promotes better dispersion of species in the electrolyte and facilitates their incorporation during the electrodeposition process, thereby improving the coating structure. Sodium acetate, in turn, helps maintain the pH at approximately 4, stabilizes the complexed species at higher concentrations and contributes to a more stable deposition process.^29,30^ The corresponding complexation reaction is shown below. Although the present study focuses primarily on the electrochemical corrosion protection performance of the coatings, further studies involving adhesion and mechanical characterization will be necessary to assess the industrial applicability of these coatings more comprehensively.

$${\mathrm{Sn}}^{2+}+{2C}_{6}H_{11}O_{7}^{-}\to \mathrm{Sn}{\left(C_{6}H_{11}O_{7}\right)}_{2}$$

Sodium lauryl sulfate, a surfactant, enhances the wettability of the substrate surface, promoting the formation of a more uniform coating with fewer pores. It also facilitates the dispersion of electrolyte particles, supports the growth of coating grains, and inhibits the adherence of gas bubbles, such as H_2_, to the substrate, which can lead to defects in the deposited film.^31^

The tin–graphene oxide composite coatings were electrodeposited like that of pure tin. Under the electrochemical conditions employed, graphene oxide (GO) may undergo partial electrochemical reduction, leading to the formation of partially reduced graphene oxide (rGO), as reported in previous studies.^32–34^ The processes involved can be represented schematically as

$${\mathrm{Sn}}^{2+}+{2e}^{-}\to \mathrm{Sn}$$

$$GO+xe^{-}+yH^{+}\to rGO+H_{2}O$$

These reactions represent a simplified schematic description of the possible processes occurring during electrodeposition. The extent of GO reduction within the coating was not directly determined in this study and would require further characterization, such as Raman spectroscopy or detailed XPS analysis.

The deposits generally resembled pure tin in their appearance, particularly in color. Plates coated with films 1, 5, 7, 10, and 11 exhibited a silver-white shade, whereas plates 2, 6, 8, and 9 appeared darker and more grayish. In terms of uniformity and brightness, plate 1 showed the highest visual quality, while the other coatings displayed varying degrees of defects, such as pores and cracks. Plates 9, 6, and 3 showed considerable heterogeneity and a scaly appearance of the deposits. These undesirable features are likely related to the applied current densities or potential instabilities of the plating bath. For example, higher concentrations of graphene oxide may negatively affect the bath stability.

These plates were also subjected to potentiodynamic polarization and electrochemical impedance tests under the same conditions used for the tin coatings. The corresponding polarization curves and impedance diagrams for each sample were obtained, and the stabilized open-circuit potentials (OCPs) are presented in Table 11.

**Table 11:** Stabilized Open Circuit Potentials (OCP) of Sn–GO-Coated Plates after 24 h of Exposure to a 3.5% (W/V) NaCl Corrosive Solution

Plate	Open Circuit Potential (V)
1	–0.646
2	–0.680
3	–0.499
4	–0.713
5	–0.691
6	–0.591
7	–0.567
8	–0.667
9	–0.689
10	–0.644
11	–0.611

Figure 11 shows the Nyquist diagrams of mild steel plates coated with electrodeposited Sn–GO. Plate 7 exhibits the largest capacitive loop, with the semicircle presenting the greatest diameter among the samples. This indicates that the coating on plate 7 has the highest charge transfer resistance, suggesting superior corrosion resistance compared to the other coatings.

**Figure 11:**
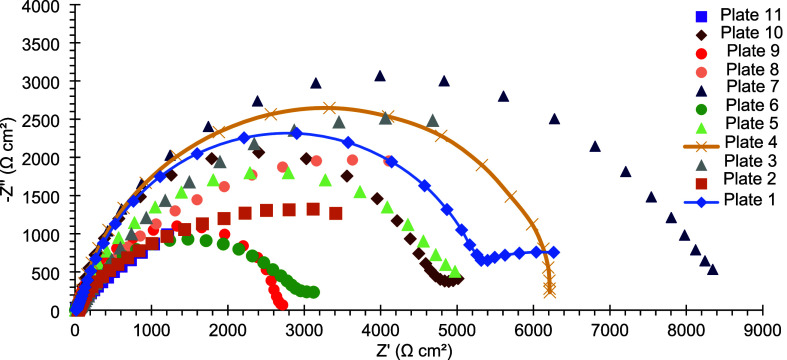
Nyquist diagram of mild steel plates coated with electrodeposited Sn–GO after 24 h of exposure to a 3.5% (w/v) NaCl corrosive solution.

Figures 12 and 13 present the Bode diagrams of the same coated plates. Regarding the impedance modulus, plate 7 shows the highest value at the lowest frequency (f = 0.01 Hz), further indicating a superior corrosion resistance (Table 12). Additionally, plate 7 exhibits one of the highest maximum phase angles, consistent with behavior approaching that of an ideal capacitor. In contrast, the phase angle curve of plate 1 displays two closely spaced maxima, suggesting the presence of two coupled electrochemical processes with similar time constants.

**Figure 12:**
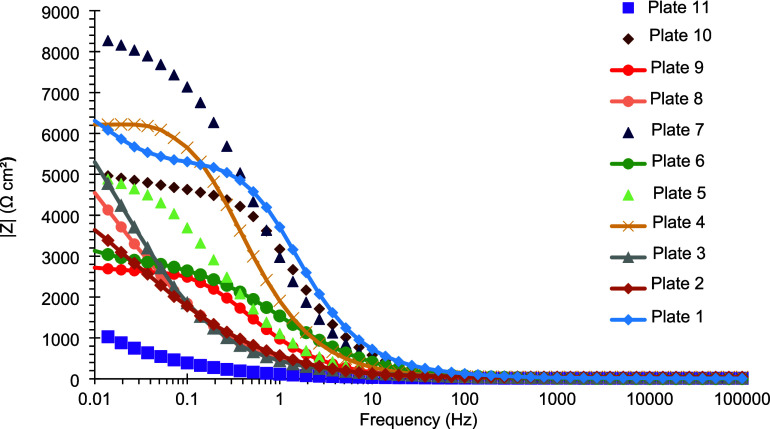
Bode diagram showing the impedance modulus of electrodeposited Sn–GO coatings after 24 h of exposure to a 3.5% (w/v) NaCl corrosive solution.

**Figure 13:**
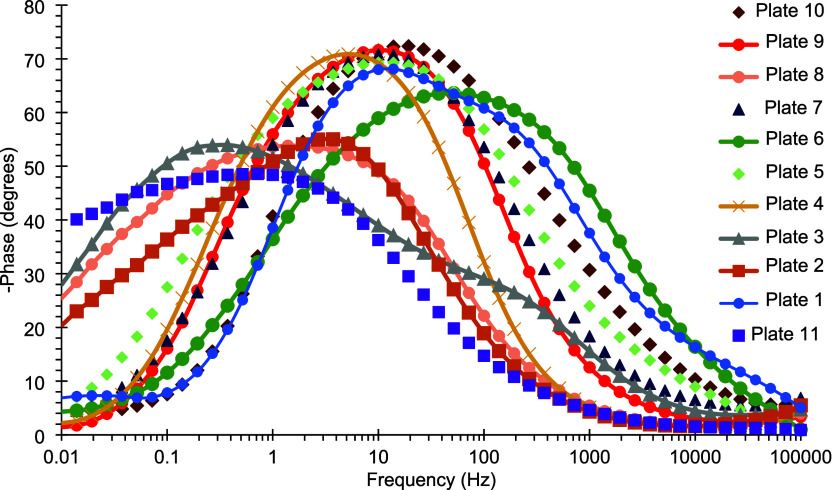
Bode diagram showing the phase angle of electrodeposited Sn–GO coatings after 24 h of exposure to a 3.5% (w/v) NaCl corrosive solution.

**Table 12:** Impedance Modulus at 0.01 Hz and Maximum Phase Angle of Sn–GO-Coated Plates aAfter 24 h of Exposure to a 3.5% (W/V) NaCl Corrosive Solution

Plate	|Z|_0.01 Hz_ (Ω cm^2^)	–Φ_max_ (degrees)
1	6309	68
2	3640	55
3	5298	54
4	6219	71
5	4999	69
6	3130	64
7	8363	71
8	4548	54
9	2718	72
10	5029	72
11	1196	48

As previously discussed, the cathodic curves exhibit a large region of nearly constant cathodic current density, indicative of a diffusion-controlled process. Therefore, the Tafel extrapolation method was not applied, and the polarization data were analyzed qualitatively. In the curves for plates 1 and 7, shown in Figures 14 and 15, a decrease in the current density is observed in the anodic branch, likely reflecting the electrodeposition conditions used for these coatings. However, beyond this region, the current density rises again for both plates, suggesting a possible coating breakdown.

**Figure 14:**
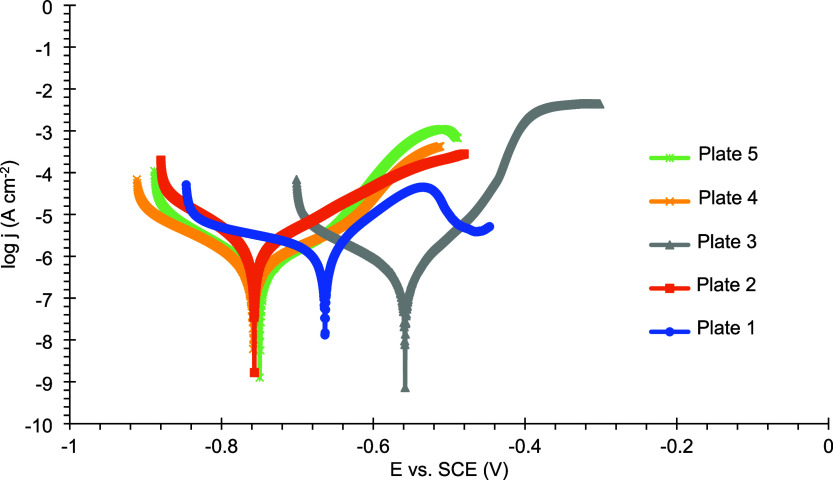
Polarization curves of plates 1–5 coated with Sn–GO after 24 h of exposure to a 3.5% (w/v) NaCl corrosive solution.

**Figure 15:**
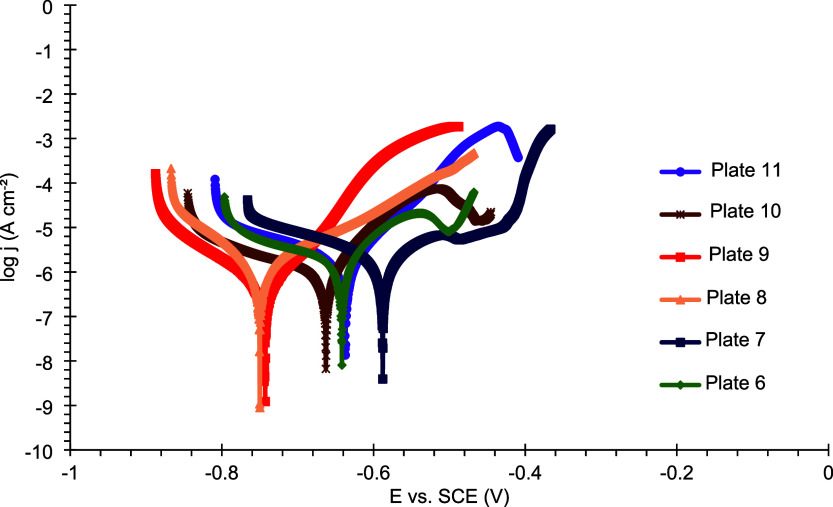
Polarization curves of plates 6–11 coated with Sn–GO after 24 h of exposure to a 3.5% (w/v) NaCl corrosive solution.

Similarly, to the approach adopted for Sn coatings, the impedance modulus data of the Sn–GO samples were analyzed using the Action Stat software to evaluate the statistical significance of the factors affecting them, using the Plackett–Burman statistical model. The effects were calculated as the difference between the average responses at the high (+1) and low (−1) levels of each factor according to the table below (Table 13).

**Table 13:** Plackett-Burman Design Matrix and Experimental Responses for Sn–GO

Run	A	B	C	|Z|_0.01 Hz_ (Ω cm^2^)
1	10.0 (+1)	0.125 (−1)	10 (−1)	6309
2	2.0 (−1)	0.625 (+1)	30 (+1)	3640
3	6.0 (0)	0.375 (0)	20 (0)	5298
4	2.0 (−1)	0.625 (+1)	10 (−1)	6219
5	10.0 (+1)	0.625 (+1)	30 (+1)	4999
6	6.0 (0)	0.375 (0)	20 (0)	3130
7	10.0 (+1)	0.125 (−1)	30 (+1)	8363
8	2.0 (−1)	0.125 (−1)	30 (+1)	4548
9	10.0 (+1)	0.625 (+1)	10 (−1)	2718
10	6.0 (0)	0.375 (0)	20 (0)	5029
11	2.0 (−1)	0.125 (−1)	10 (−1)	1196

Due to the absence of replicates in the factorial portion, the statistical significance of the effects was evaluated using Lenth’s method. The pseudo standard error (PSE), margin of error (ME), and the simultaneous margin of error (SME) were determined at a 95% confidence level, as illustrated in Figure 16.

**Figure 16:**
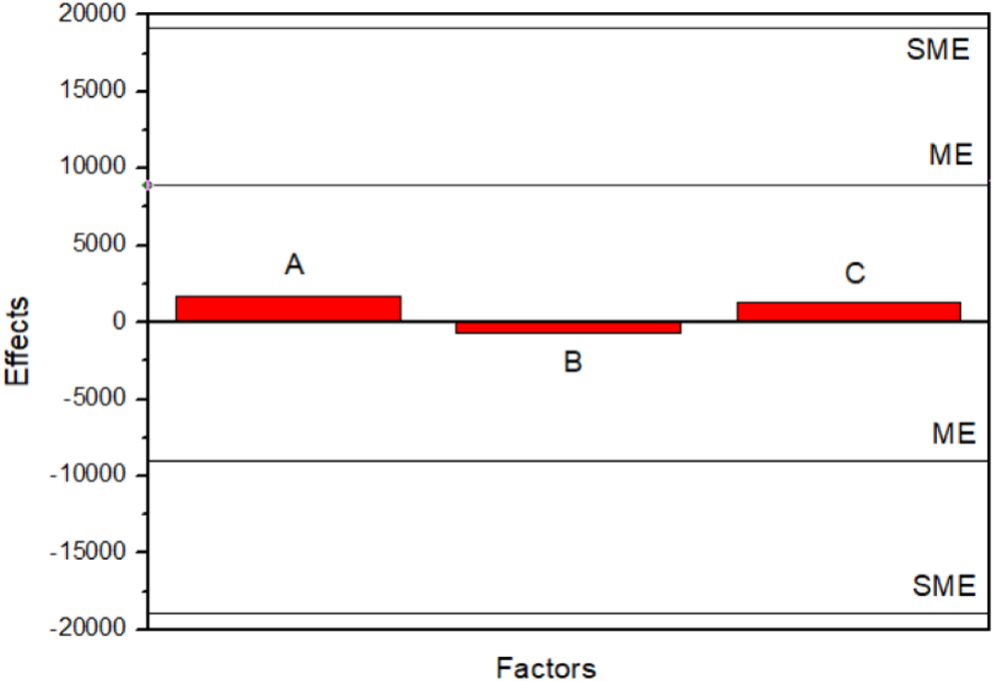
Half-normal plot of the effects for Sn–GO.

The estimated effects for all factors and their interactions were below the margin of error. Specifically, the main effects of current density (A = 1696.72), GO concentration (B = −710.22), and deposition time (C = 1276.28), as well as all interaction terms, were not statistically significant. This result is confirmed by the half-normal plot, in which all effects fall within the nonsignificant region.

The absence of statistically significant effects indicates that, within the investigated experimental domain, none of the evaluated parameters—current density, GO concentration, or deposition time—exerts a significant influence on the impedance modulus of the Sn–GO coatings.

From a physical perspective, this suggests that the corrosion resistance of the coatings is relatively insensitive to variations in the electrodeposition parameters within the selected ranges. This behavior may indicate that the coating formation process occurs within a stable regime in which small changes in the operating conditions do not significantly affect the electrochemical performance.

The responses at the central point (5298, 3130, and 5029) are comparable in magnitude to those of the factorial runs, indicating no significant curvature in the response surface. This observation supports the use of a first-order model to describe the system in the investigated region.

Although interaction effects were estimated, it is important to note that Plackett–Burman designs are primarily intended for screening main effects, and interaction terms may be aliased. Therefore, the interpretation of these interactions should be approached with caution.

Since no statistically significant effects were identified, it was not possible to establish a predictive model for optimization. Therefore, the optimal conditions were selected based on the experimental response values.

The condition that yielded the highest impedance modulus (|Z| = 8363 Ω cm^2^) was considered the most favorable within the studied range, as higher impedance values are associated with improved corrosion resistance. This response was obtained for plate 7, corresponding to a GO concentration of 0.125 g L^–1^, a current density of j = 10.0 mA cm^–2^, and a deposition time of 30 min.

Although no statistically significant factors were identified in either study, this result is still meaningful as it indicates that current density, GO concentration, and deposition time do not play a significant role within the investigated experimental window.

This finding allows a reduction in the number of variables to be considered in future studies. Further investigations using more advanced experimental designs, such as full factorial or response surface designs, are recommended to explore potential nonlinear effects, interactions, and other parameters that may govern coating performance.

In this context, when the optimal condition reported by Gupta and Srivastava (2018) (0.375 g L^–1^ GO, j = 6.25 mA cm^–2^, and t = 20 min) was approximately reproduced in this study, represented by plates 3, 6, and 10, an average value of 4486 Ω cm^2^ with a standard deviation of 1182 Ω cm^2^ was obtained, which is significantly lower than that reported by Gupta and Srivastava.^11^ This discrepancy may arise from differences in experimental conditions. However, by increasing the current density and deposition time, a superior coating was achieved with a lower GO concentration (0.125 g L^–1^). This behavior may be likely due to the more intense deposition conditions, which promote the formation of a more consolidated film and reduce galvanic corrosion associated with high GO concentrations. This results in a more effective barrier against the corrosive environment, although such interpretations should be made with caution due to the lack of statistical significance.

These findings highlight the importance of varying deposition conditions in screening studies—an aspect not explored by Gupta and Srivastava, whose analysis focused solely on GO concentration. Within this framework, the corrosion resistance may be influenced by the deposition parameters, depending on the investigated range. Consequently, a GO concentration of 0.125 g L^–1^ emerged as the most favorable condition within the studied experimental domain, potentially leading to a thicker, more structured coating and thereby enhancing its barrier effect compared to the previous approach.

The morphology and microstructure of this coating were further examined by using scanning electron microscopy (SEM). Figure 17 shows an SEM micrograph of a representative area of the best-performing composite coating.

**Figure 17:**
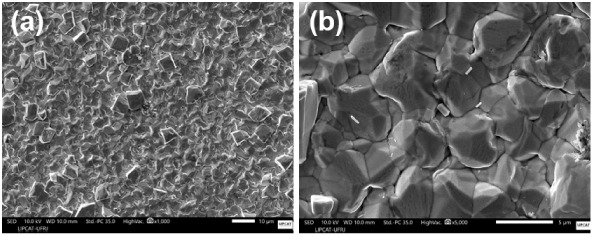
(a) Low-magnification and (b) high-magnification SEM micrographs of the Sn–GO coating on plate 7.

The morphology of the Sn–GO coating is polyhedral, featuring larger, well-defined grains with flat faces and continuous coverage. The incorporation of graphene oxide (GO) results in a more homogeneous structure, promoting the coalescence of faceted crystals and reducing surface roughness, which indicates a modification in the nucleation and growth mechanism. No pores or cracks were observed, demonstrating preserved structural integrity. The grains exhibit less irregularity, as GO serves as a nucleating agent, producing a more compact and uniform coating compared to pure Sn. This enhanced microstructure accounts for the observed improvement in corrosion resistance, providing a more effective barrier against corrosive species. The elemental composition of the composite coating in the same region was further analyzed using SEM-EDS, as shown in Figure 18.

**Figure 18:**
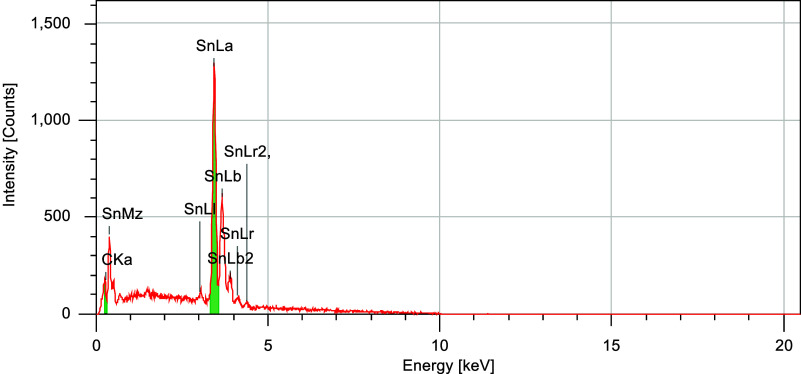
SEM-EDS analysis of the Sn–GO composite coating on plate 7.

SEM-EDS analysis of the surface confirmed the presence of carbon alongside tin, indicating the successful deposition of the composite. The composition was determined to be 99.15% Sn and 0.85% C. These values are consistent with literature reports on metal–graphene oxide composite coatings, suggesting that the detected carbon originates from the incorporated GO rather than from extrinsic impurities.^35^

### 3.5 Performance Validation of Sn and Sn–GO Coatings

In this study, the deposition was repeated under the best-performing conditions for the Sn–GO coating, and duplicate samples were prepared. The replicate plates produced under these conditions exhibited the expected silvery-white appearance, particularly plate PO3. However, some cracks and heterogeneities were observed.

Electrochemical impedance and polarization tests were also performed for validation, as this condition had been tested only once in the original experimental plan. A comparison of the samples in terms of open-circuit potentials, Nyquist plots, and Bode plots is presented in Table 14 and Figures 19–21, respectively.

**Table 14:** Stabilized Open Circuit Potentials (OCP) of the Duplicate Sn and Sn–GO-Coated Plates at the Best-Performing Deposition Conditions after 24 h of Exposure to a 3.5% (m/v) NaCl Solution

Plate	Open Circuit Potential (V)
Sn PO6	–0.642
Sn PO7	–0.684
Sn–OG PO1	–0.557
Sn–OG PO3	–0.569

**Figure 19:**
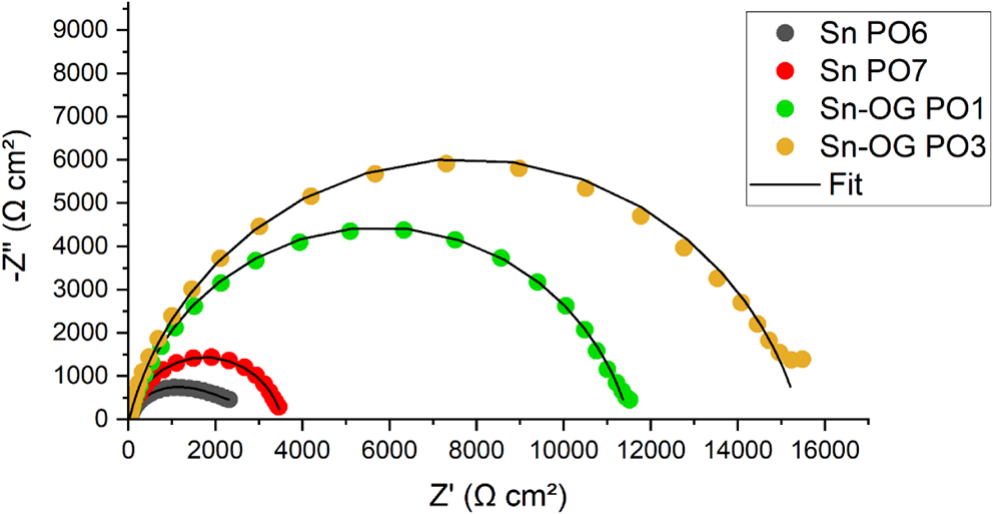
Nyquist diagram of experimental data and simulated fits for duplicate Sn and Sn–GO coatings of the best-performing after 24 h of exposure to a 3.5% (m/v) NaCl solution.

**Figure 20:**
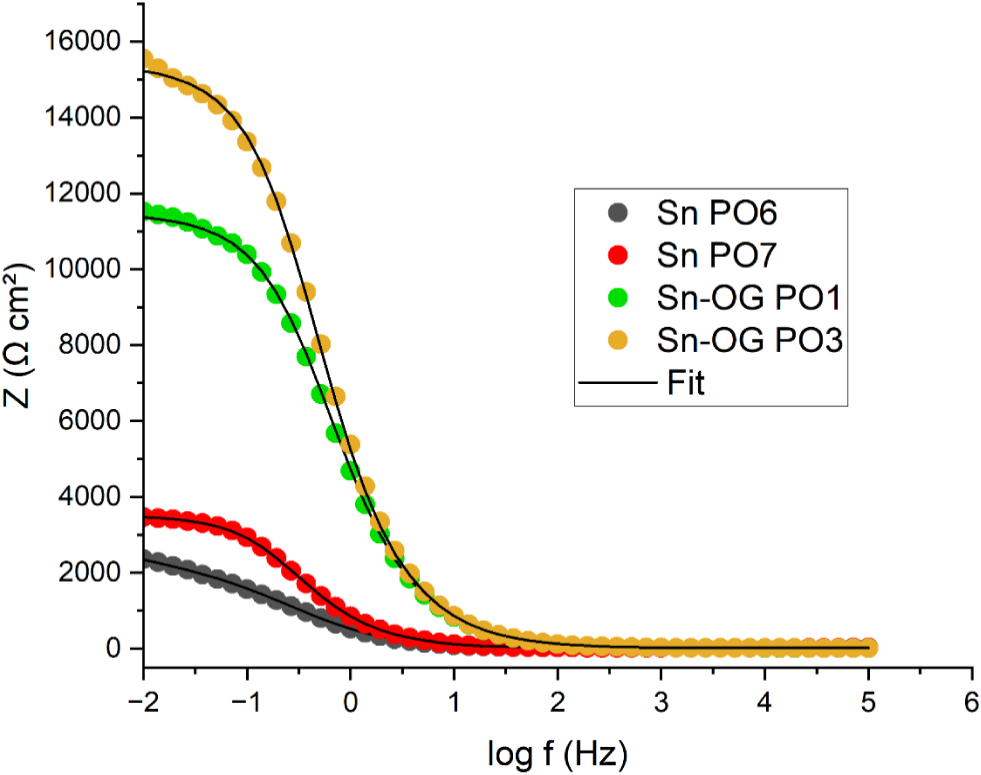
Bode diagram (impedance modulus) showing experimental data and simulated fits for duplicate Sn and Sn–GO coatings of the best-performing after 24 h of exposure to a 3.5% (m/v) NaCl solution.

**Figure 21:**
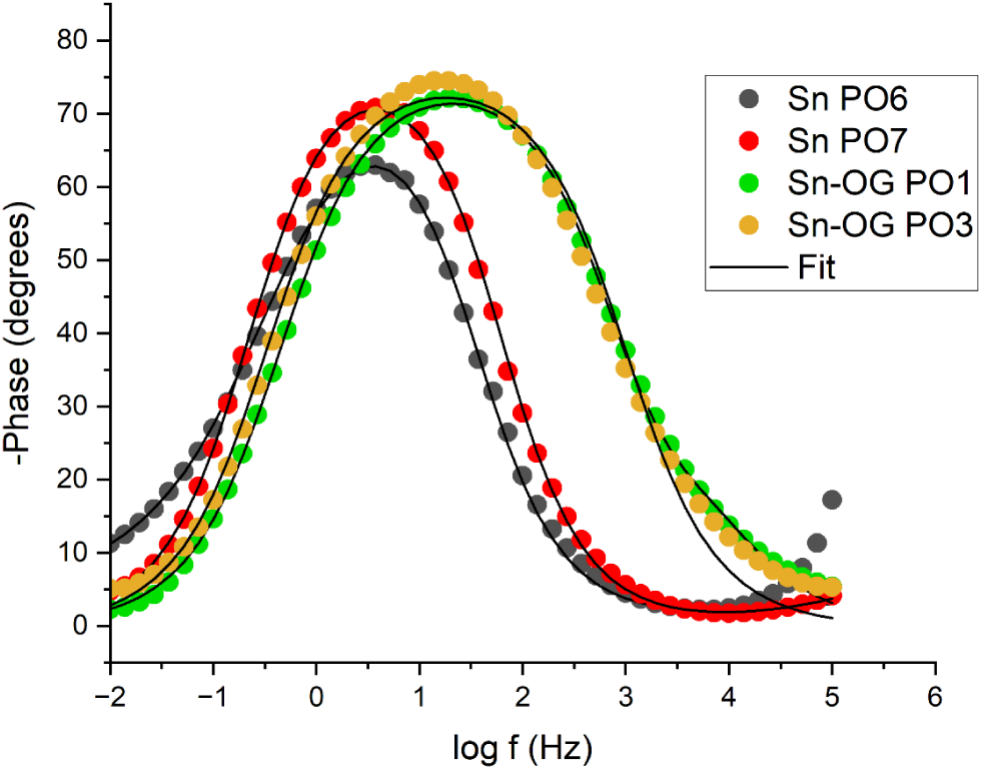
Bode diagram (phase angle) showing experimental data and simulated fits for duplicate Sn and Sn–GO coatings of the best-performing after 24 h of exposure to a 3.5% (m/v) NaCl solution.

In the Nyquist (Figure 19) and Bode (Figures 20 and 21) diagrams for the duplicate samples under the best-performing conditions—tin (Sn PO6 and Sn PO7) and the composite (Sn–OG PO1 and Sn–OG PO3)—the charge transfer resistance and apparent electric double-layer capacitance were calculated using ZSimDemo 3.60 software, based on the equivalent circuit shown in Figure 22. The simulated curves (Fit) were plotted alongside the experimental data for comparison. Visual inspection indicated good agreement between the fitted curves and experimental points, suggesting that the chosen circuit adequately represents the electrochemical system.

**Figure 22:**
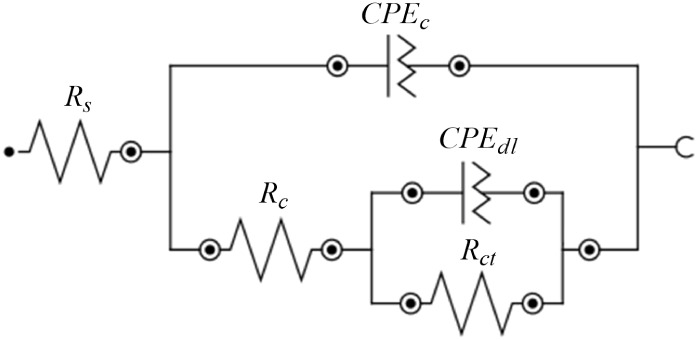
Equivalent circuit used for the duplicate samples of the best-performing Sn and Sn–GO coatings.

The values of the equivalent circuit elements calculated by the software are presented in Table 15.

**Table 15:** Electrochemical Parameters of the Equivalent Circuit Elements for Duplicate Sn and Sn–GO-Coated Plates of the Best-Performing after 24 h of Exposure to a 3.5% (m/v) NaCl Solution

Coating	*R*_s_ (Ω cm^2^)	*R*_ct_ (Ω cm^2^)	*C*_dl_ (μF cm^–2^)	*n*	*R*_c_ (Ω cm^2^)	*C*_c_ (μF cm^–2^)	*n*	χ^2^
Sn PO6	5.83	3630	285.6	0.881	23.8	263.8	0.252	4.32 × 10^–3^
Sn PO7	5.80	4038	177.9	0.891	21.7	10.4	0.472	1.64 × 10^–4^
Sn–OG PO1	14.1	11451	12.7	0.878	19.4	19.7	0.878	2.78 × 10^–4^
Sn–OG PO3	17.4	14847	9.2	0.980	29.7	31.7	0.844	4.65 × 10^–3^

Here, *R*_ct_ is the charge transfer resistance, *R*_c_ is the coating resistance, *R*_s_ is the solution resistance, *C*_dl_ is the apparent electric double-layer capacitance, and *C*_c_ is the apparent coating capacitance, since the capacitive response is described by a constant phase element (*n* ≠ 1). These apparent capacitances were calculated following the approach proposed by Hsu and Mansfeld,^36^ according to

$$C=Y_{0}{\left(2\pi f_{\max}\right)}^{n-1}$$

where *Y*_0_ is the admittance parameter of the constant phase element (CPE) (*S* s^*n*^ cm^–2^), *f*_max_ is the frequency at which the imaginary impedance of the Nyquist plot (−Z″) reaches its maximum, and *n* is the ideality parameter of the capacitor. The parameter *n* reflects how closely the coating behaves as an ideal capacitor, with values near 1 indicating a more homogeneous coating and behavior approaching that of an ideal capacitor. The parameter χ^2^ serves as a statistical indicator of the quality of the equivalent circuit fit, with lower χ^2^ values indicating a smaller fitting error for the selected model.

To describe the system, the equivalent circuit shown in Figure 22, typical of coatings, was employed, with the corresponding element values listed in Table 15. In this circuit, *CPE*_dl_ and *CPE*_c_ correspond to the constant phase elements associated with the electric double layer and coating, respectively. The Nyquist diagram (Figure 19) shows a larger capacitive loop for the Sn–GO duplicates compared to the Sn duplicates, indicating superior corrosion resistance of the composite relative to pure tin. This is supported by the charge-transfer resistance (*R*_ct_) values in Table 15, which are nearly 4 times higher for the nanocomposite coatings. Similarly, the apparent electric double-layer capacitance (*C*_dl_) is lower for the Sn–GO coatings, suggesting a reduced likelihood of corrosion. Lower capacitance implies less charge accumulation on the coating surface, decreasing the probability of corrosion reactions. The χ^2^ parameter ranged from 10^–3^ to 10^–4^, well within the acceptable range, indicating an excellent fit of the equivalent circuit to the experimental data.

The coating resistance (*R*_c_) and apparent coating capacitance (*C*_c_) provide additional insight into the structure, thickness, and dielectric behavior of the films. The Sn–OG PO3 sample exhibited the highest *R*_c_, indicating a thicker, more cohesive coating. This indicates that GO incorporation enhances the barrier properties and modifies the dielectric behavior of the film, as reflected in the *C*_c_ values. Conversely, an abnormally high capacitance was observed for the Sn PO6 sample; this should be interpreted cautiously, as the low phase exponent (*n* = 0.25) indicates strongly nonideal behavior. Under these conditions, the apparent capacitance no longer accurately represents the constant phase element (CPE), making physical interpretation of *C*_c_ unreliable. The low *n* value suggests pronounced coating heterogeneity, possibly due to pores or high surface roughness, consistent with the lower *R*_ct_ for this sample. A low *n* was also observed for Sn PO7, in agreement with SEM analyses that showed better surface homogeneity in the GO-containing coatings.

The equivalent circuit used is similar to that proposed by Gupta and Srivastava (2018), as the electrochemical system studied here shares analogous characteristics.^11^ The main difference is the replacement of the ideal capacitor with a CPE in parallel with the coating resistance, better representing surface irregularities and heterogeneities. The *C*_dl_ values for the Sn–GO duplicates (12.7 and 9.2 μF cm^–2^) were significantly lower than those reported by Gupta and Srivastava (43.3 μF cm^–2^), indicating a reduced tendency for redox reactions at the electrode/electrolyte interface. The parameters *R*_c_ and *C*_c_ were not reported individually in their study; however, the interfacial polarization resistance (69206 Ω cm^2^) was much higher than that observed in the present work (11525 and 15552 Ω cm^2^). This discrepancy may arise from differences in experimental conditions, such as the immersion time before electrochemical impedance spectroscopy (EIS), substrate pretreatment, and, more importantly, the GO synthesis method, which can lead to variations in sheet size and, consequently, coating performance. In this study, EIS measurements were performed at the open circuit potential (OCP) after 24 h of exposure of each sample to the NaCl solution. In contrast, Gupta and Srivastava conducted their measurements after only 1 h.

In this work, an acceptable relative standard deviation was observed for the impedance modulus of each duplicate (Table 16), indicating satisfactory reproducibility despite the inherent variability of coating systems. It is worth noting that relatively high deviations, in some cases reaching or exceeding 50%, have been reported in EIS measurements of coatings and are associated with factors such as coating and bath heterogeneity, thickness variations, and application conditions.^37,38^ In particular, small differences in coating morphology or local defects, for example, can lead to large fluctuations in impedance values, especially at low frequencies, where the measurement is more sensitive to barrier properties.^37,38^ Therefore, the magnitude of the observed deviations should be interpreted within the expected experimental uncertainty for EIS analyses of coatings and does not compromise the reliability of the overall trend discussed in this study, which is consistent with literature reports for Sn and Sn–GO systems,^11^ reinforcing that the conditions identified as the best remain the best-performing, despite the observed variability.

**Table 16:** Impedance Modulus, Maximum Phase Angle, and Relative Standard Deviation of Duplicate Sn and Sn–GO-Coated Plates under the Best-Performing Conditions after 24 h of Exposure to a 3.5% m/v NaCl Solution

Coatings	|Z|_0.01 Hz_ (Ω cm^2^)	Relative Standard Deviation (%)	–Φ_max_ (degrees)
Sn–OG PO1	11525	21	72
Sn–OG PO3	15552		74
Sn PO6	2364	27	63
Sn PO7	3470		71

Considering these aspects, one can note that the Sn–GO composites exhibited larger capacitive loops in the Nyquist diagram (Figure 19), higher impedance modulus at 0.01 Hz, and phase angles closer to 90° in the Bode diagrams (Figures 20 and 21). These results corroborate the equivalent circuit analysis, confirming the superior corrosion resistance of the Sn–GO coatings.

Regarding the polarization curves, all coatings exhibited profiles similar to those previously observed. However, only the Sn–OG PO1 and Sn–OG PO3 coatings showed a significant reduction in anodic current density, supporting the hypothesis that incorporating graphene oxide enhances barrier properties. This is further corroborated by the lower anodic current densities of the composites compared to the pure Sn coating, as well as the shift of the corrosion potential toward more positive values, as shown in Figure 23.

**Figure 23:**
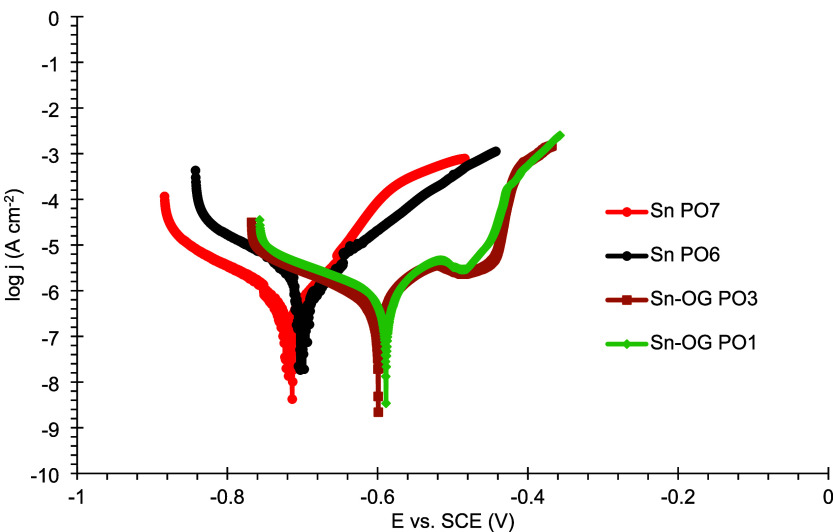
Polarization curves of duplicate plates coated with Sn and Sn–GO under the best-performing conditions after 24 h of exposure to a 3.5% m/v NaCl solution.

The duplicate Sn–GO composite coatings exhibited greater corrosion resistance under the best-performing conditions compared to the Sn duplicates under their respective conditions. This improvement can be attributed to the presence of graphene oxide, which enhances the texture and morphology of the deposit grains, thereby increasing their barrier properties relative to the pure metallic coating. The average impedance data for the Sn and Sn–GO coatings under the best-performing conditions, calculated from Sn–OG PO1, Sn–OG PO3, Sn PO6, and Sn PO7, are presented in Table 17.

**Table 17:** Average Electrochemical Parameters from Impedance Measurements and Equivalent Circuit Modeling for the Sn and Sn–GO-Coated Plates with the Best Performance after 24 h of Exposure to a 3.5% (m/v) NaCl Solution

Coating under the best-performing conditions	*R*_s_ (Ω cm^2^)	*R*_ct_ (Ω cm^2^)	*C*_dl_ (μF cm^–2^)	*R*_c_ (Ω cm^2^)	*C*_c_ (μF cm^–2^)	|Z|_0.01 Hz_ (Ω cm^2^)	–Φ_max_ (degrees)
Sn	5.82	3834	231.7	22.8	137.1	2917	67
Sn–GO	15.8	13149	10.9	24.6	25.7	13538	73

To better contextualize the performance of the Sn–GO composite coatings developed in this study, the electrochemical results were compared with values reported in the literature for other advanced anticorrosive coatings (Table 18). The impedance modulus and charge transfer resistance obtained for the best Sn–GO coating were significantly higher than those observed for pure Sn coatings and are comparable to values reported for other graphene-based and metal–GO composite systems.

**Table 18:** Comparison of Charge Transfer Resistance (*R*_ct_) Values Reported in the Literature for Graphene-Based and Hybrid Anticorrosive Coatings on Metallic Substrates and the Sn–GO Coating Developed in This Work

Coating	Substrate	Surface	*R*_ct_ (kΩ cm^2^)	Reference
Sn–GO	Mild steel	Sn only	3.83	This work
		Sn–GO	13.15	
Graphene Coating	Cu	Cu	1.71	Hasan et al
		Cu–graphene	3.95	
Graphene coating	Cu	Cu	2.95	Prasai et al.
		Cu–graphene	10.1	
Zn–GO	Mild steel	Zn only	0.297	Rekha M Y and Chandan Srivastava
		Zn–GO	0.705	
Zn–GO	Cu	Zn only	0.282	Ruiqian Li et al.
		Zn–GO	1.474	

Graphene-based protective films have been widely reported to significantly enhance the corrosion resistance of metallic substrates due to their excellent barrier properties and high chemical stability.^8,39^ In these studies, graphene coatings improved corrosion resistance by up to 3.4 times compared with uncoated systems, which is consistent with the enhancement observed in the present work.

Gupta and Srivastava (2018) investigated the effect of incorporating different concentrations of graphene oxide into tin coatings electrodeposited on steel and identified an optimal GO content associated with enhanced corrosion resistance. However, their study was conducted under fixed electrodeposition conditions (j = 6.25 mA cm^–2^ and t = 20 min) and without prior optimization of the deposition parameters for pure tin coatings, which limits a direct and systematic comparison between the pure Sn and Sn–GO systems.

In contrast, the present study first investigated the electrodeposition parameters for pure tin coatings and subsequently evaluated GO incorporation under different conditions. The results indicated that the highest corrosion resistance was achieved for plate 7, corresponding to a GO concentration of 0.125 g L^–1^, a current density of 10.0 mA cm^–2^, and a deposition time of 30 min.

Increasing the current density and deposition time led to improved performance at a lower GO concentration (0.125 g L^–1^). These results suggest that electrodeposition parameters may influence the coating microstructure and the distribution of GO within the metallic matrix; however, no statistically significant factors were identified within the investigated ranges. The selected deposition conditions may favor the formation of a more compact and homogeneous film, which could reduce the likelihood of localized galvanic effects associated with excessive GO content and contribute to a more effective barrier against corrosive environments.

Similarly, Zn–GO composites have been investigated on different substrates, showing increases in charge transfer resistance (*R*_ct_) of up to 5.2 times compared to pure Zn coatings, further highlighting the beneficial role of graphene oxide in enhancing the corrosion protection performance of metallic coatings.^40,41^

It should be noted that direct comparisons of *R*_ct_ values reported in different studies must be made with caution, as these parameters are strongly influenced by experimental factors such as electrolyte composition, immersion time, substrate preparation, coating thickness, and measurement conditions.^37^ In particular, the immersion time before EIS measurements in the present study differs from that adopted by Gupta and Srivastava, which may partially account for the observed differences in performance. Despite these variations, the Sn–GO composite coatings developed in this work exhibited competitive electrochemical behavior compared with other graphene-based systems reported in the literature. Notably, the optimized coating (plate 7) showed a significant increase in charge transfer resistance compared with pure tin coatings.

Furthermore, an important advantage of the present system is that enhanced corrosion resistance was achieved with relatively low GO concentrations (0.125 g L^–1^) and a scalable electrodeposition process. These results highlight the potential of Sn–GO coatings as an effective strategy for improving the corrosion resistance of carbon steel substrates.

## 4 Conclusions

XRD characterization of the synthesized graphene oxide confirmed the presence of its characteristic diffraction peaks, while FTIR analysis revealed the vibrational bands associated with oxygen-containing functional groups, confirming the successful formation of the graphene oxide.

The electrodeposition parameters were investigated by varying the current density and deposition time for metallic tin coatings, and the current density, deposition time, and GO concentration for the tin–graphene oxide (Sn–GO) composite coatings. Among the evaluated conditions, the Sn–GO coating obtained on plate 7 exhibited the best performance, corresponding to a GO concentration of 0.125 g L^–1^, a current density of 10 mA cm^–2^, and a deposition time of 30 min.

Morphological observations indicated that the Sn–GO coatings exhibited superior physical characteristics compared with pure tin coatings, including improved uniformity, greater thickness, enhanced brightness, and a silvery-white appearance. Electrochemical impedance spectroscopy and polarization measurements demonstrated that the Sn–GO coatings, particularly at low GO concentrations, showed significantly improved anticorrosive performance compared with pure tin coatings after 24 h of exposure to a 3.5% NaCl solution.

Under the selected conditions, duplicate samples presented an average charge transfer resistance of 3834 Ω cm^2^ for the pure tin coating and 13149 Ω cm^2^ for the Sn–GO composite coating, confirming the enhanced corrosion resistance resulting from the incorporation of graphene oxide.

These results are consistent with previous studies reporting that incorporating graphene oxide into metallic coatings improves corrosion resistance through its barrier effect and its influence on coating morphology and microstructure. Overall, the Sn–GO composite coating shows strong potential as an alternative to conventional tin coatings for developing corrosion-resistant protective systems in industrial applications.

These findings highlight the potential of Sn–GO composite coatings as an efficient and scalable strategy to enhance the corrosion resistance of tin-based protective systems.
